# The Active Phytohormone in Microalgae: The Characteristics, Efficient Detection, and Their Adversity Resistance Applications

**DOI:** 10.3390/molecules27010046

**Published:** 2021-12-22

**Authors:** Chun Wang, Mei Qi, Jiameng Guo, Chengxu Zhou, Xiaojun Yan, Roger Ruan, Pengfei Cheng

**Affiliations:** 1 College of Food and Pharmaceutical Sciences, Ningbo University, Ningbo 315211, China; 2111091090@nbu.edu.cn (C.W.); 206003454@nbu.edu.cn (M.Q.); guojiam98@163.com (J.G.); zhouchengxu@nbu.edu.cn (C.Z.); 2 Key Laboratory of Applied Marine Biotechnology of Ministry of Education, Ningbo University, Ningbo 315211, China; 3 Center for Biorefining and Department of Bioproducts and Biosystems Engineering, University of Minnesota-Twin Cities, Saint Paul, MN 55108, USA; ruanx001@umn.edu

**Keywords:** microalgae, phytohormone, analysis, abiotic stress

## Abstract

Phytohormones are a class of small organic molecules that are widely used in higher plants and microalgae as chemical messengers. Phytohormones play a regulatory role in the physiological metabolism of cells, including promoting cell division, increasing stress tolerance, and improving photosynthetic efficiency, and thereby increasing biomass, oil, chlorophyll, and protein content. However, traditional abiotic stress methods for inducing the accumulation of energy storage substances in microalgae, such as high light intensity, high salinity, and heavy metals, will affect the growth of microalgae and will ultimately limit the efficient accumulation of energy storage substances. Therefore, the addition of phytohormones not only helps to reduce production costs but also improves the efficiency of biofuel utilization. However, accurate and sensitive phytohormones determination and analytical methods are the basis for plant hormone research. In this study, the characteristics of phytohormones in microalgae and research progress for regulating the accumulation of energy storage substances in microalgae by exogenous phytohormones, combined with abiotic stress conditions at home and abroad, are summarized. The possible metabolic mechanism of phytohormones in microalgae is discussed, and possible future research directions are put forward, which provide a theoretical basis for the application of phytohormones in microalgae.

## 1. Introduction

With the rapid development of the global economy, energy consumption of various countries has risen sharply. This has expanded the demand to find new sources for renewable, clean energy [1]. Microalgae are considered a potential source of renewable energy due to their high oil content and short growth cycle [2]. Microalgae can use light energy, CO_2_, and inorganic nutrients to accumulate bioactive substances; the photosynthetic efficiency is 10–50 times higher than that of terrestrial plants [3]. However, the extensive accumulation of lipids during large-scale cultivation of microalgae is often offset by the rapid growth of algal cells. The separation of lipids from the mass of algal cells causes microalgae bioenergy to become difficult to compete with traditional fossil fuels [4]. Therefore, it is urgent to find an effective and low-cost technology to improve the oil production per unit of algal cells.

Phytohormones are a class of small, organic, molecular compounds that participate in the regulation of cell life processes and can adapt to their own growth in response to external, adversarial conditions [5]. In general, as signaling molecules, phytohormones are divided into five categories: auxin (IAA), cytokinin (CK), abscisic acid (ABA), ethylene (ETH), and gibberellin (GA). In addition, brassinosteroids (BRs), jasmonate (JAs), and salicylic acid (SA) are also, currently, classified as plant hormones. Although there are few reports on microalgae phytohormones, they can promote lipid accumulation in algal cells without affecting cell metabolism and growth [6]. Furthermore, phytohormones can increase cell growth and metabolites and lipid accumulation by improving the activity of related enzymes in microalgae [7]. Therefore, adding phytohormones on the basis of traditional culture may improve the efficiency of biofuel utilization and thereby help to reduce production costs [8].

Phytohormones are also related to cell stress. Abiotic stress, such as high light intensity, high temperature, heavy metals, and salt stress, can induce energy storage in microalgae cells, resulting in different degrees of lipid accumulation [9]. However, due to the simultaneous production of a large number of reactive oxygen species (ROS) by algal cells under stress conditions, it can seriously affect the growth, metabolism, and antioxidant capacity of microalgae, which can limit the efficient accumulation of energy storage and can significantly reduce the growth, development, and metabolism of the algae [10]. By adding exogenous phytohormones, such as brassinosteroids or stratolactone, this can prevent the outbreak of reactive oxygen species by activating antioxidant enzymes and antioxidants and thus can limit cell damage induced by abiotic stress [11]. Therefore, it may be possible to regulate the growth and metabolism of microalgae by combining plant hormones and abiotic stresses. This may not only improve photosynthetic efficiency and promote lipid accumulation but also adapt to the stressful environmental conditions posed by large-scale processing.

However, the metabolic process of phytohormones in microalgae is not as clear as in higher plants. The main obstacle is that the content of phytohormones in microalgae cells is low, and the analysis and detection methods are complex [12]. Little is known about the regulatory role of phytohormones in microalgae. It is still necessary to refer to the path conduction and mechanism of action of higher plant hormones. Therefore, this review discusses the characteristics and determination methods of microalgae plant hormones, and it explains the possible mechanism of microalgae plant hormones in response to abiotic stress, so as to provide a theoretical basis for microalgae oil production and other functional applications.

## 2. Characteristics of Plant Hormones

Phytohormones are small chemical messenger compounds that are widely found in higher plants and in microalgae. They are trace organic substances, synthesized in plants, and they play an important regulatory role in the whole process of plant life activities. In plants, plant hormone content is very low, but it is involved in almost every process that regulates growth and development, including regulating their own growth and metabolism and regulating the adaptation to adversities in the environment [6].

Previous studies have shown that auxin (IAA) has a great influence on the physiological and biochemical processes of higher plants; exogenous IAA can accelerate fruit development and induce drought tolerance [13]. CK has many physiological functions in plants, including stimulating cell division and differentiation, promoting biogenesis and chloroplast differentiation, and preventing leaf senescence [14]. ABA can induce leaf droop, stomatal closure, and senescence; hinder the synthesis of nucleotides and proteins; and inhibit plant growth [15]. ETH is a gaseous plant hormone that not only can regulate plant growth, development, and senescence but also can develop tolerance to environmental factors from biotic stress (pathogen invasion) and abiotic stress (including drought, high salinity, and cold) [16].

At present, there are many studies on plant hormones that regulate higher plants, but there are relatively few studies on the function of plant hormones in microalgae [17]. *Charophyte* algae are the closest relatives of land plants [18,19], and traces of plant hormone synthesis and signal channels have been found in the whole genome of some microalgae [20]. Therefore, in a sense, the phytohormone biosynthetic pathway of higher plants may originate from single-celled microalgae [21], and the effects of phytohormones on microalgae may be similar to those of higher plants. According to the characteristics of plant hormones in higher plants, the research and utilization of microalgae phytohormones may provide new ideas for the improvement of microalgae biofuels and the anti-stress activity of high-value products [22]. 

### 2.1. Auxin (IAA)

The biological functions of auxin in algae are similar to those in higher plants [23]. Indole-3-acetic acid is the most important auxin in plant cells. IAA, IBA, IPK, and IAM have been found in 46 microalgae belonging to Cyanophyta and Chlorophyta [24]. Auxin plays a multifaceted role in the growth and metabolism of microalgae. Even very low concentrations of auxin can stimulate growth and increase biomass and biosynthesis of high-value biomolecules [25,26,27], and higher concentrations of auxin can inhibit cell growth [28].

### 2.2. Cytokinin (CK)

Cytokinin is the product of purine, and the main form is zeatin. It has two structures: cis and trans. In microalgae, it is mainly the cis structure [29]. CK can induce microalgae cell division, enhance photosynthetic pigment accumulation, and improve photosynthetic efficiency, and thus promote biomass accumulation [30]. Cytokinins exist at low concentration during the dark period and at relatively high concentration in the light period [31]. Under unfavorable environmental conditions, CKs have protective effects on the physiological activities of microalgae, especially photosynthesis.

### 2.3. Abscisic Acid (ABA)

Abscisic acid is a sesquiterpene compound with 15 carbons that exists mostly in aging organs or tissues and plays a physiological role by inhibiting cell growth [32]. Likewise, ABA can help microalgae enter a dormant state under adverse conditions, cope with environmental pressure, and enhance the stress resistance of microalgae [33,34]. For example, in *Haematococcus pluvialis*, ABA can inhibit the growth of algal cells and trigger the transition of cells from active phase to stationary phase [35].

### 2.4. Ethylene (ETH)

Ethylene is an unsaturated hydrocarbon containing two carbon atoms, which can regulate the growth and development of microalgae and which plays an important role in higher plants [36,37]. However, there are few studies on ethylene in microalgae. Although it is believed that ethylene is a growth inhibitor, more and more evidence shows that when the ethylene concentration is within a certain range, it can also promote growth and biosynthesis [38,39]. For example, 0.05 mL/L ethylene significantly increased astaxanthin accumulation in *Haematococcus pluvialis*, while 0.1 mL/L ethylene can inhibit it. The addition of ethephon (an ethylene release agent) will promote the increase in proline and saturated fatty acid content, but the content of citric acid and unsaturated fatty acid will decrease [40].

### 2.5. Others

Gibberellin is a kind of shell shirtene compound that can be divided into seven forms, such as GA_1–7_, according to the difference in double bonds and hydroxyl groups of its structure [41]. Gibberellin can participate in cell elongation and affect the growth and metabolism of microalgae cells by regulating carbon metabolism [42,43,44].

Brassinosteroid (BRS) is a new plant hormone that can regulate cell division and cell elongation, improve cell antioxidant capacity, promote cell absorption of nutrients, and synthesize proteins, nucleic acids, carbohydrates, and photosynthetic pigments required for growth [45,46]. In addition, BRs also play an important role in the resistance of microalgae to abiotic stresses (such as heavy metals, high/low temperature, high salt) [47]. BRs can also cooperate with other plant hormones, such as auxin, to promote cell division and the synthesis of metabolites [48].

Salicylic acid (SA) is a phenolic compound synthesized in plants. Its regulatory effect on microalgae is mainly reflected in the impact on the antioxidant system. SA can promote the accumulation of antioxidant substances by promoting the generation of H_2_O_2_ and improving the expression of carotenoid-related genes, which has a protective effect on abiotic stress of algal cells [49]. It can also promote carbohydrate and protein degradation by affecting the transcription of related enzymes, thereby increasing lipid accumulation in microalgae [50,51].

Jasmonic acids (JAs) mainly include jasmonic acid and methyl jasmonate, which are mainly involved in signal transduction under abiotic stress in microalgae. Similar to SA, JAs can increase the expression of carotene-related genes in microalgae and promote the accumulation of antioxidant substances such as astaxanthin and β-carotene [7]. In a word, microalgae phytohormones may play a role in regulating the growth, development, and metabolism of microalgae (Table 1).

## 3. Biosynthesis of Plant Hormones

**Figure 1:**
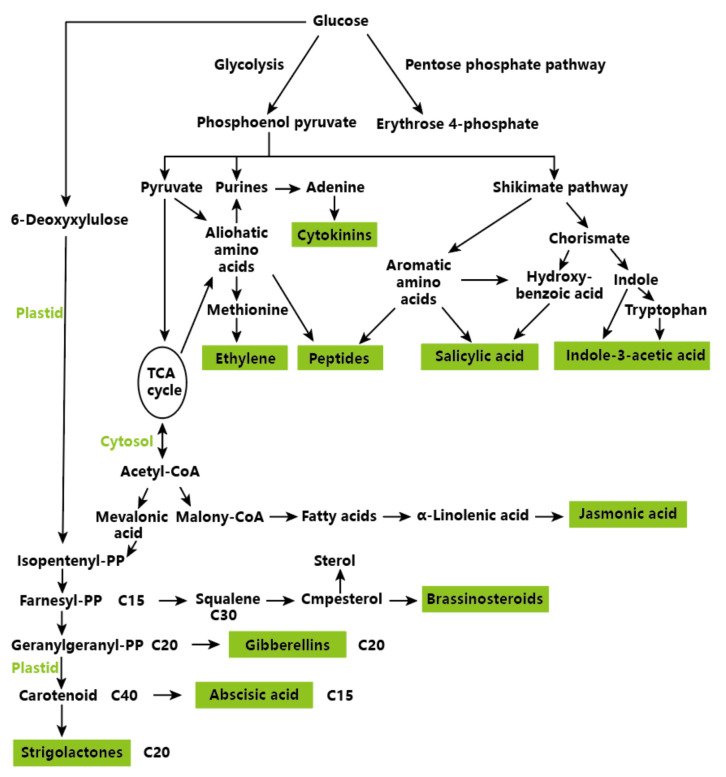
Biosynthesis Pathways of Plant Hormones in Higher Plants. Adapted with the permission from Springer (2012) [52].

In general, the higher plant hormone biosynthesis pathway has been relatively detailed. Auxin, cytokinin, ethylene, and salicylic acid are mainly produced by glucose through glycolysis, as shown in Figure 1. After glycolysis, adenine can be produced and directly produce cytokinin, while ethylene comes primarily from methionine. Indole-3-acetic acid comes from tryptophan, and indole-3-acetic acid can also be directly synthesized through precursor indole. C-40 carotenoids in the plastid can be oxidized to produce different fragments, which will be further metabolized into active hormones, mainly abscisic acid and strigolactone (Figure 1).

The expression of plant hormone biosynthetic genes was detected in most tissues to varying degrees. Except ETH, SA, and ABA, all other hormones are produced in various forms. IAA, CK, GA, JA, BR, SL, and peptide hormones can exist through dozens of different structural forms, which makes plant hormones specific and selective, so different chemical forms of hormones can trigger different reactions [53].

**Figure 2:**
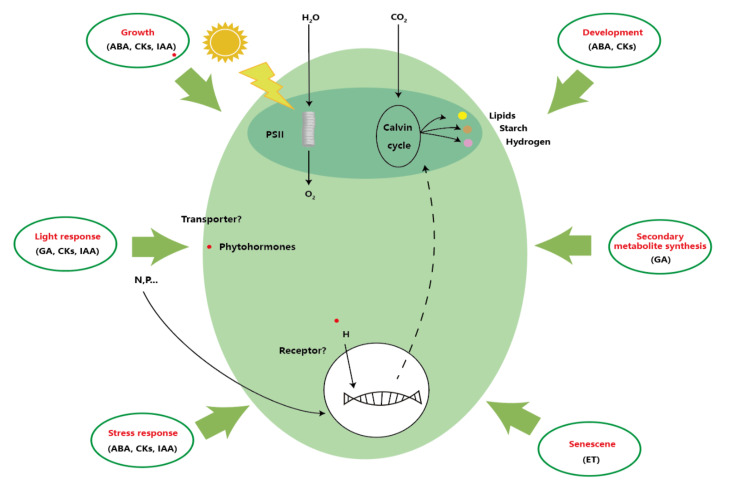
Potential strategies for manipulating plant hormone metabolism. Adapted with the permission from Elsevier (2015) [21].

Nevertheless, the research on plant hormone function in microalgae started relatively late, and many were derived or deduced from the correlation between exogenous higher plant hormones and microalgae. However, the synthesis and metabolic regulation of plant hormones in microalgae may be different from those in higher plants, as shown in Figure 2 [21]. For instance, there are IAA synthesis homologous genes similar to those in higher plants in the genome of *Ectocarpus siliculosus*. However, the results of gene chip analysis show that the expression of auxin-induced gene *EsGRP1* is inversely proportional to cell differentiation in different morphological mutants. Therefore, the effect of auxin in *Ectocarpus siliculosus* may be different from that in terrestrial plants on cell differentiation localization and cell signal pathway induction. IAA regulates growth and development in higher plants, but in *Ectocarpus siliculosus*, it seems to play a regulatory role in transmitting cell location information and inducing a signaling pathway different from that known in terrestrial plants [54]. The mechanism of auxin action between unicellular green algae and higher plants is also fundamentally different. Green algae have simpler auxin signaling elements and pathways, suggesting that complex auxin signaling occurs between the evolution of single-cell microalgae to early terrestrial plants [55]. In addition, higher plants are directly involved in the regulation of auxin under heavy metal stress [56], and exogenous auxin addition to higher plants can also interact with miRNA to regulate [57]. In algae, the role of auxin is mainly related to the detoxification of ROS. Even without heavy metal stress, auxin can stimulate the antioxidant system and reduce the levels of peroxide and hydrogen peroxide [58]. In higher plants, ABA is derived from isoprenoids synthesized by 1-deoxyxylulose 5-phosphate (MEP) pathway in plastids. In comparison, CKs are synthesized by isoprenoids, and isoprenoids are produced by the methoxylvalerate pathway, which plays a role in cytoplasm. Most algae only express enzymes in the MEP pathway and use it for isoprenoid biosynthesis, while marine diatoms (*P. tricornutum*, *T. pseudonana*, and *F. cylindrus*) and brown algae (*E. siliculusus*) seem to use both pathways at the same time, and homologues of known higher plant ABA glycosyltransferases have not been found in marine microalgae. Similarly, despite the lack of glucose-based transferases from terrestrial plants, different glucose-based CKs were detected in marine algae [59]. Nevertheless, the study of the synthesis and metabolism of plant hormones in microalgae cells can still refer to the corresponding situation of higher plants.

## 4. Determination and Analysis of Plant Hormones

Different from other intracellular active substances, plant hormones have many types, low content, large differences in structural characteristics, complex components, and a variety of derivatives and metabolites. Therefore, accurate, reliable, and convenient quantitative analysis of plant hormones is necessary for the study of plant hormone physiological functions.

### 4.1. Physical Chemistry Method

Due to the low and mostly unstable plant hormone content, it is suitable to use high sensitivity, simple, and rapid detection methods. The commonly used gas chromatography and high-performance liquid chromatography can better meet these conditions.

#### 4.1.1. High-Performance Liquid Chromatography (HPLC)

High-performance liquid chromatography (HPLC) uses an aqueous solution as the mobile phase, which has the advantages of high sensitivity and good repeatability. It is the main method for accurate determination of endogenous hormones. Liquid chromatography-mass spectrometry combines the high separation ability of high-performance liquid chromatography for complex samples. Its advantages of high selectivity and high sensitivity of mass spectrometry effectively reduces the absorption of ultraviolet compounds and shows high sensitivity to plant hormones [12,60]. A liquid chromatography-tandem mass spectrometry (LC-MS/MS) method was used to concentrate and purify different groups of plant hormones from plant samples by solid phase extraction (SPE) pretreatment, and quantitative measurement was carried out by selective reaction monitoring (SRM). The results showed that the SPE-LC-MS/MS method was very effective for the simultaneous determination of GA_3_, IAA, and ABA [61].

#### 4.1.2. Gas Chromatography (GC)

Gas chromatography has the advantages of high sensitivity and good separation and selection performance, and it is a commonly used method for the determination of plant hormones. However, the GC method requires the analyte to have a low polarity and gasification temperature. Therefore, in addition to ethylene, plant hormones generally need to be derivatized to generate corresponding derivatives to carry out GC analysis. The derivatization process is more complicated, which increases the workload of sample pretreatment. In recent years, more and more studies have been conducted on gas chromatography-mass spectrometry (GC-MS), which has become an important means for quantitative analysis of plant endogenous hormones [62]. At present, the GC method is more accurate and reliable, but the pretreatment workload is large, and the equipment is expensive, and the maintenance cost is also high.

### 4.2. Electrochemical Analysis

Compared with the above methods, electrochemical analysis is simple and inexpensive. Some studies have found that the nature and pH of the background solution of plant hormones have a great impact on the determination of results, and the situation is more complex in plant samples, so electrochemical methods are less used for the determination of actual samples [63,64]. In recent years, newly developed electrochemical biosensors have shown good development potential in the analysis and detection of plant hormones due to their unique advantages of wide operation range, good selection performance, and high sensitivity [65].

### 4.3. Others

#### 4.3.1. Biological Assay

A biological assay was the earliest method used to determine the endogenous hormones in plants, and the content is estimated by the intensity of various specific reflections produced by endogenous hormones. Although this method can reflect the physiological activity of hormones, the specificity is poor, and the influencing factors such as auxin analogues and antagonists in plants need to be removed, and the determination process is complex [66]. Therefore, the bioassay method is mainly used for qualitative analysis of plant hormones, or it is combined with physical and chemical analysis for quantitative determination [67,68].

#### 4.3.2. Immunoassay

An immunoassay is a detection method for qualitative or quantitative analysis of analytes, using antibodies as analytical reagents and specific binding characteristics between antibodies and antigens. However, this method still has problems, such as unstable operation, large interference, and poor repeatability. It has not been widely used in the analysis and determination of plant hormones [69,70,71].

## 5. Application of Plant Hormones

### 5.1. Physiological Response

#### 5.1.1. The Effect of Plant Hormones on Growth and Primary Metabolites

Plant hormones such as auxin, jasmonic acid, and salicylic acid act on algae growth in a concentration-dependent manner [72]. The treatment of green algae with 0.1 μM IAA/IBA or 1 μM NAA and PAA can increase the content of soluble protein, chlorophyll, carotenoid, and monosaccharides in primary metabolites. The biological activity of natural auxin IAA is the highest, and NAA is a synthetic auxin with the lowest activity [58,73]. It is speculated that auxin may stimulate the activity of photosynthesis by increasing the content of chlorophyll and activating the cellular redox system, thereby promoting growth [74]. It was also found that the content of chlorophyll a and b supplemented with seven auxin precursors and analogues increased by 213–273%, and the carotenoid content of *C. pyrenoidosa* increased by 164–258% [75].

Using 10^−5^ to 10^−4^ M high concentration of JA treatment can lead to a decrease in cell number, photosynthetic pigments, monosaccharides, and extracellular protein levels. Low concentrations (10^−8^ to 10^−6^ M) of JA induce an increase in cell number, photosynthetic pigments, and monosaccharide content [76]. SA also has a dose-dependent stimulation effect, which can increase the cell number, dry weight, and primary metabolite content, such as chlorophenol, carotenoid, monosaccharide, soluble protein, DNA, and RNA [77]. The CK biosynthesis of microalgae mainly depends on nitrogen. It enhances nitrogen assimilation by activating glutamate dehydrogenase, stimulates the carbon cycle of Calvin cycle reaction, and finally increases the RNA, protein level, and the peptide content in microalgae [78]. Exogenous cytokinins can promote the accumulation of endogenous brassinosteroids [79], and BRs have the greatest impact on the growth and metabolism of algae within 24 to 36 h after treatment [80]. It was reported that gibberellin is mainly involved in cell elongation and expansion, but not in cell division [13]. External use of gibberellin can significantly shorten the lag period and activate cell division and growth in the log phase.

#### 5.1.2. Effects of Plant Hormone on Oil Products

Microalgae lipid is a promising renewable raw material for biodiesel and renewable diesel production. Although higher growth rates have a negative impact on lipid accumulation, oil production increases as biomass increases [81]. In addition, IAA can enhance photosynthetic activity by increasing chlorophyll content and increasing the content of monounsaturated fatty acids [82]. IAA and DAH can increase the content of polyunsaturated fatty acids by 56% and 59% [26]. Another study showed that GA_3_ promotes the growth and lipid accumulation of *C. pyrenoidosa* by increasing esterase activity and mediating intracellular carbon distribution. The maximum lipid content (292.3 mg/g) and lipid productivity (17.1 mg/L/day) can be reached with the concentration of 20 mg/L GA_3_, and higher levels of GA_3_ show a 1.6-fold increase in unsaturated fatty acid content [83]. ABA, brassinolide (BL), and IAA have a positive role in promoting lipid biosynthesis in *S. quadricauda*. Fatty acid accumulation is observed at 100 μM ABA [84], and a supplement of cytokinin can also increase lipid productivity to 63.14 mg L^−1^ d^−1^ [85]. In addition, supplements of ethephon (an ethylene release agent) to the *C. vulgaris* culture will increase the levels of proline and saturated fatty acids but decrease the levels of citrate and unsaturated fatty acids [86].

### 5.2. Stress Resistance Response of Plant Hormones

The photosynthetic efficiency of microalgae is 10–50 times that of terrestrial plants, while the theoretical bio–oil production of some oil-producing microalgae is 10–100 times that of terrestrial plants [87], so microalgae are considered to be a potential renewable raw material for future new energy and chemicals [88]. However, the raw material cost of mass production of microalgae is too high, and it is not practical to use microalgae to produce biofuel in the short term. Park et al. used phytohormones to realize the economic feasibility of large-scale production of microalgae and found that compared with the cost of additional carbon sources and other substances, the addition of phytohormones not only helps to reduce production costs but also improves the efficiency of biofuel utilization [8]. Abiotic stresses (including nitrogen, phosphorus, salt, high light, high temperature, and other stresses) can promote lipid accumulation to varying degrees but also have a serious impact on the growth, metabolism, and antioxidant capacity of microalgae [89]. In addition, plant hormones or plant growth regulators, such as melatonin, betaine, fulvic acid, and BL, have certain regulatory effects on the growth metabolism and antioxidant capacity of microalgae.

#### 5.2.1. Nutrients

Nitrogen starvation can promote lipid accumulation in microalgae cells, but low biomass can affect lipid production. This is due to the excessive amount of reactive oxygen species (ROS) that are produced by microalgae under stress and which significantly reduces the growth, development, and metabolism of algae [90,91,92,93]. The addition of exogenous plant hormones can change this phenomenon. Up-regulation of ACCase and RuBisCO in microalgae improved the quality of biodiesel upon the addition of IAA application [10], which improved the quality of biodiesel and alleviated the adverse effects of nitrogen stress. After exogenous addition of IBA and NAA, *Scenedesmus SDEC-8* and *Chlorella sorokiniana SDEC-18* not only maintained the biomass concentration of the microalgae, alleviating the damage of nitrogen stress, but also improved the lipid yield of algal cells [94]. At the same time, after the addition of cytokinin, the photosynthetic efficiency of the algal cells increased, and the biomass and lipid yield of the microalgae increased by 50% and 60.7%, respectively [86]. After 48 h of cultivation under nitrogen-restricted conditions, the biomass of *Scenedesmus quadricauda* added with 2 μM ABA increased by 2.1 times, which may be a potential strategy for high-efficiency microalgae cultivation for biofuel production [95]. In addition, salicylic acid and jasmonic acid can induce the activity of the antioxidant system, enhance the accumulation of soluble proteins, free proline synthesis, and improve the tolerance of plants to abiotic stress, thereby enhancing the adaptation to stress conditions, such as nitrogen stress [96].

#### 5.2.2. Heavy Metals

Metal pollution in the environment is a major issue worldwide [97]. Increasing the tolerance of plants to heavy metals and improving the growth and adaptability of plants under metal stress provides an ecologically safe and economically efficient means for bioremediation of heavy metals [98].

**Figure 3:**
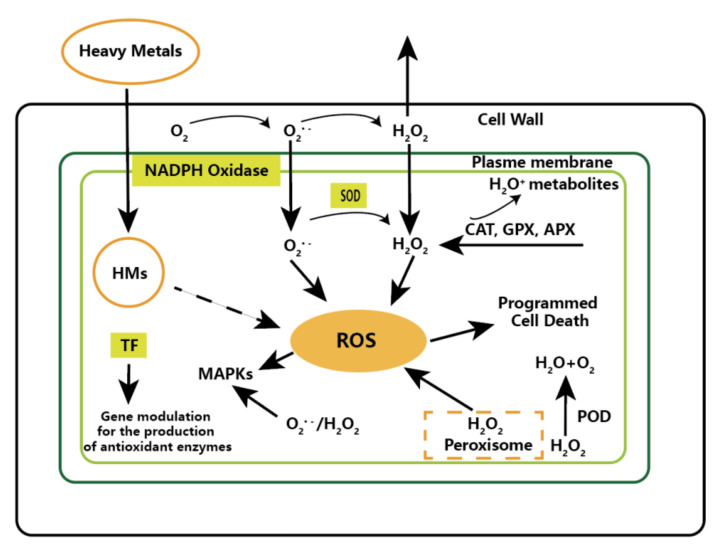
Graphical representation of reactive oxygen species (ROS) in heavy metal signal transduction. Adapted with the permission from Elsevier (2016) [99].

Heavy metal toxicity is mainly reflected in the production of reactive oxygen species, and reactive oxygen species can accelerate lipid peroxidation, thereby affecting cell membrane fluidity and permeability. Reactive oxygen species can trigger gene activation involved in inducing different metabolic pathways to deal with heavy metal toxicity (Figure 3) [99]. Algae tolerance to heavy metals is based on two mechanisms. The first is through the adsorption of heavy metals into the cell surface. For example, exogenous auxin can improve the transport of metals through the algal cell membrane and its cellular translocation. The second mechanism is to prevent the biological use of toxic metals through complexation [100].

Auxin, cytokinin, gibberellin, and spermidine can alleviate stress symptoms by inhibiting heavy metal biosorption, thus restoring algae growth and primary metabolite levels [101]. More results show that auxins (IAA, IBA, NAA, and PAA) have an effect on the algae *C. vulgaris* exposed to heavy metal (Cd, Cu, and Pb) stress and non-enzymatic (ascorbic acid and glutathione) antioxidant systems, thereby inhibiting lipid peroxidation and hydrogen peroxide accumulation [58]. Exogenous cytokinins can protect proteins and other components required for photosynthesis, and they significantly reduce the damage by heavy metals to *Chlorella vulgaris* and *Acutodesmus obliquu* [102]. When algal cells are stressed by cadmium, copper, or lead, cytokinin also reduces the toxicity of heavy metals by inhibiting ROS formation [103]. GA3 has a function for protecting the microalgae *C. vulgaris* through the action of cell count and accumulation of protein, photosynthetic pigments, and monosaccharides to resist the stress of Pb and Cd [101]. Exogenous brassinolide can also alleviate the inhibition of heavy metals on microalgae by reducing the accumulation of heavy metals in cells, stimulating the production of ABA and IAA, and improving the levels of chlorophyll, sugar, and proteins [104].

#### 5.2.3. Light and Temperature

Light is the main limiting factor of microalgae productivity. Low light limits algal cell growth, while high light inhibits photosynthetic activity. Under low or high light, salicylic acid can increase the activity of superoxide dismutase by 3.3 to 4.5 times and the activity of ascorbate peroxidase (APX) by 7.1 to 15.5 times, respectively. Methyl jasmonate can increase catalase activity under strong light and APX activity under weak light. At low concentrations, SA and MJ can be used to induce the production of secondary carotenoids, while at high concentrations, they can inhibit astaxanthin accumulation by scavenging free radicals, or they can increase the production of primary carotenoids [105]. Exogenous melatonin treatment also prevents reactive oxygen species outbreaks and can limit abiotic stress-induced cell damage by activating antioxidant enzymes and antioxidants [106].

Temperature affects all metabolic processes and improves the heat tolerance strategy of microalgae in different seasons. The effects of adding glycine betaine at low temperature has been reported. Compared with the control treatment at 26 °C, the addition of exogenous glycine betaine at low suboptimal temperatures improved photosynthesis by enhancing the expression of genes encoding Rubisco and also improved lipid productivity [107].

## 6. Future Perspectives and Conclusions

At present, the research on regulating the accumulation of energy storage substances in microalgae by adding plant hormones has focused on oil-producing microalgae with nitrogen limitation; the effects of stress conditions on microalgae have been less studied. Plant hormones play a positive regulatory role by reducing oxidative stress. The combination of exogenous plant hormones, and abiotic stress conditions, can achieve a simultaneous yield increase in microalgae biomass and energy storage material. In addition, there are at present more and more studies of microalgae phytohormones, but they mainly focus on the detection methods of phytohormones and their roles in algae cells. However, there are a few studies on the metabolic pathways of microalgae phytohormones and the interaction between hormones. The regulatory mechanism in microalgae is not completely clear, and there is a lack of research on endogenous plant hormones in microalgae. Therefore, it is suggested that future research may focus on:

(1) in-depth study of the molecular mechanism of plant hormones that regulate the metabolism of energy storage substances in microalgae;
(2) the response and regulation mechanism of the metabolic process of microalgae, under stimulation of plant hormones and systematically studied by multi-omics combined analyses, and;
(3) screening of cheaper and more effective plant hormones for metabolic regulation of microalgae under other stress conditions, such as high salinity and high light intensity.

## Figures and Tables

**Table 1:** Typical plant hormones in microalgae. Adapted with the permission from Elsevier (2020) [22].

	Structural Formula	Targets Promoted	Reference
Auxins	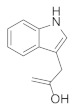	Growth Lipid Chlorophyll-a Soluble proteins	[25] [26] [27]
Cytokinin	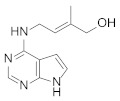	Biomass Lipid Carbohydrate Proteins	[29] [30]
Abscisic acid	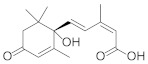	Growth β-carotene Lipid Carotenoids	[35]
Ethylene	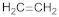	A-tocopherol C-aminobutyric acid Proline Astaxanthin	[37] [38] [40]
Gibberellin A_4_	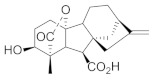	Biomass Lipid Carbohydrate Proteins	[42] [43] [44]
Brassinolide	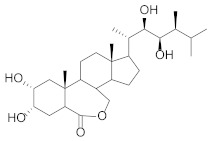	Protein Nucleic acid Carbohydrate Photosynthetic pigments	[46] [47]
Jasmonoyl-isoleucine	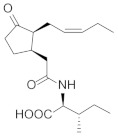	Carbohydrates Proteins Lipids Carotenoids	[51,52,53]
Salicylic acid	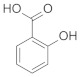	Astaxanthin β-carotene Carbohydrates Proteins	[50] [51]

## Data Availability

Data available on request.
